# The complete mitochondrial genome of *Anoplistes halodendri* (Coleoptera: Cerambycidae)

**DOI:** 10.1080/23802359.2020.1850218

**Published:** 2020-12-24

**Authors:** Bairong Lin, Rong Deng, Jiayi Ma, Xiaoqian Weng, Xiong Guan

**Affiliations:** a College of Plant Protection, Key Laboratory of Biopesticide and Chemical Biology (Ministry of Education), Fujian Agriculture and Forestry University, Fuzhou, PR China; b College of Forestry, Fujian Agriculture and Forestry University, Fuzhou, PR China; c Key Laboratory of Integrated Pest Management in Ecological Forests, Fujian Province University, Fujian Agriculture and Forestry University, Fuzhou, PR China

**Keywords:** Complete mitochondrial genome, *Anoplistes halodendri*, phylogenetic analysis

## Abstract

In this study, we report the complete mitochondrial genome of *Anoplistes halodendri*, which covers a total of 15,697 bp in length with 28.27% GC content. The complete mitochondrial genome is composed of 12 protein-coding genes (PCGs) and also contains 22 transfer RNA genes (tRNAs) and two ribosomal RNA genes (rRNAs). Phylogenetic analysis of the *A. halodendri* with other 21 different species of Cerambycidae indicated that *A. halodendri* formed an isolated clade and belong to Cerambycinae. The results will be helpful to study the evolutionary relationship among the subfamilies of Cerambycidae.

*Anoplistes halodendri* is a species of Cerambycidae belong to Coleoptera, which is widely distributed in China, Korea, and Mongolia (Karpiński et al. 2018). The host plants of *A. halodendri* include *Tetraena mongolica*, *Lycium chinense, Hippophae rhamnoides*, *Lonicera japonica, Pyrus* spp., etc. (Wei et al. 2012; Karpiński et al. 2018). However, the study of *A. halodendri* on the level of genetic evolution was limited (Wang et al. 2019). In this study, we determined the complete mitochondrial genome of *A. halodendri* and comprehend the evolving relationship by phylogenetic analysis of *A. halodendri*.

The *A. halodendri* were collected from Lianjiang, Fujian Province, China (119° 38′ 25″E, 26° 9′ 21″N) and the specimens were stored in the Fujian Agriculture and Forestry University (TN-202,005). The total DNA extraction and purification from *A. halodendri* with TruSeq DNA Sample Preparation kit (Vanzyme, Nanjing, China) and QIAquick Gel Extraction kit (Qiagen GmbH, Hilden, Germany), respectively. To obtain high-quality short-read data, the purified DNA was quantified by Qubit (Thermo Fisher Scientific Inc., Waltham, MA) and the library was prepared with fragment of 300 bp randomly interrupted. The constructed library was sequenced with 150 bp pair-end reads on the Illumina Hiseq2500 platform according to the manufacturer’s protocol (Illumina, San Diego, CA). After quality control and filtering with Fastp software (Chen et al. 2018) of the Illumina data, a total of 53,248,050 clean reads were generated. MtioZ and metaSPAdes software (Nurk et al. 2017) were used to assembly the clean reads after quality control and then the assembly sequence was annotated by the MITOS web server (Matthias et al. 2013) based on the reference genome of *Nortia carinicollis* (GenBank accession no. MK863508). The complete mitochondrial genome of *A. halodendri* was a circular structurally covering a total of 15,697 bp in length (GenBank accession no. MT809475), the overall base composition was 39.6% A, 11.17% G, 17.1% C, and 32.2% T. And the GC content was 28.27%, as well as contains 36 unique genes, including 12 protein-coding genes (PCGs), 22 transfer RNA genes (tRNAs), and two ribosomal RNA genes (rRNAs).

To further explore the phylogenetic position of *A. halodendri*, a phylogenetic comparison was performed between the *A. halodendri* and other 21 different species of Cerambycidae. Multiple sequence alignment by the MUSCLE software (Edgar 2004), and the phylogenetic trees were inferred using IQ-TREE software (Nguyen et al. 2015) with the parameter of -bb 1000. The maximum-likelihood tree showed that *A. halodendri* formed an isolated clade and belong to Cerambycinae (Figure 1). The complete mitochondrial genomic data of *A. halodendri* will be helpful to provide favorable genetic information for realizing the evolutionary relationship between Cerambycidae and other Coleoptera insects.

**Figure 1.**
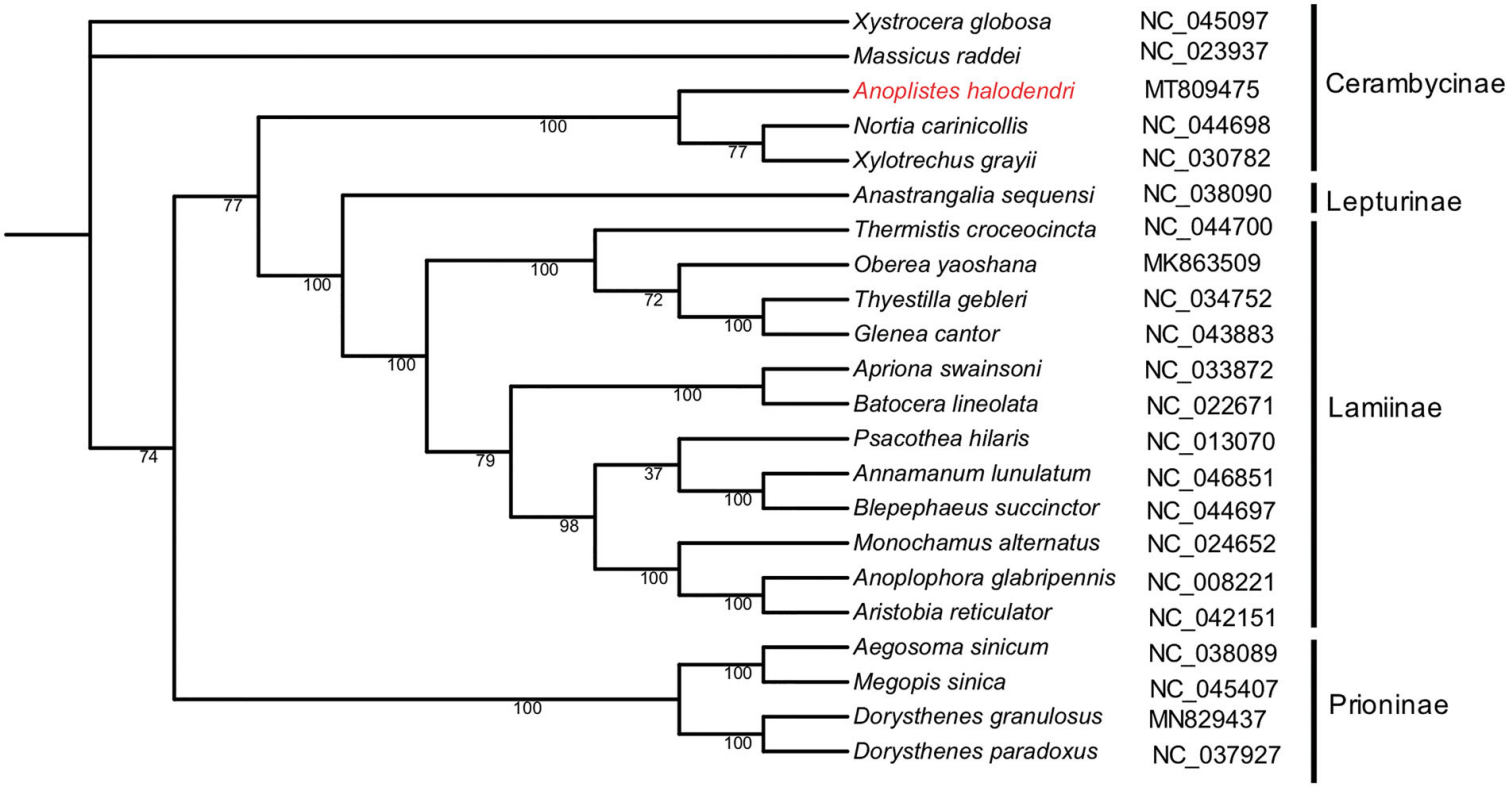
Phylogenetic relationships of the *Anoplistes halodendri* with other 21 different species of Cerambycidae based on the genome sequence. Bootstrap values are marked on the branch.

## Data Availability

The authors confirm that the data supporting the findings of this study are available within the article and are openly available in GenBank of NCBI at https://www.ncbi.nlm.nih.gov, reference number MT809475.

## References

[CIT0001] Chen SF, Zhou YQ, Chen Y, Gu J. 2018. fastp: an ultra-fast all-in-one FASTQ preprocessor. Bioinformatics. 34(17):i884–i890. 30423086 10.1093/bioinformatics/bty560 PMC6129281

[CIT0002] Edgar RC. 2004. MUSCLE: multiple sequence alignment with high accuracy and high throughput. Nucleic Acids Res. 32(5):1792–1797. 15034147 10.1093/nar/gkh340 PMC390337

[CIT0003] Karpiński L, Szczepański WT, Plewa R, Walczak M, Hilszczański J, Kruszelnicki L, Łoś K, Jaworski T, Bidas M, Tarwacki G. 2018. New data on the distribution, biology and ecology of the longhorn beetles from the area of South and East Kazakhstan (Coleoptera, Cerambycidae). Zookeys. 805(4):59–126. 10.3897/zookeys.805.29660 PMC6299065 30584393

[CIT0004] Matthias B, Alexander D, Frank J, Fabian E, Catherine F, Guido F, Joern P, Martin M, Peter FS. 2013. MITOS: improved de novo metazoan mitochondrial genome annotation. Mol Phylogenet Evol. 69(2):313–319. 22982435 10.1016/j.ympev.2012.08.023

[CIT0005] Nguyen LT, Schmidt H, von Haeseler A, Minh B. 2015. IQ-TREE: a fast and effective stochastic algorithm for estimating maximum-likelihood phylogenies. Mol Biol Evol. 32(1):268–274. 25371430 10.1093/molbev/msu300 PMC4271533

[CIT0006] Nurk S, Meleshko D, Korobeynikov A, Pevzner PA. 2017. metaSPAdes: a new versatile metagenomic assembler. Genome Res. 27(5):824–834. 28298430 10.1101/gr.213959.116 PMC5411777

[CIT0007] Wang J, Dai XY, Xu XD, Zhang ZY, Yu DN, Storey KB, Zhang JY. 2019. The complete mitochondrial genomes of five longicorn beetles (Coleoptera: Cerambycidae) and phylogenetic relationships within Cerambycidae. PeerJ. 7(2):e7633. 31534857 10.7717/peerj.7633 PMC6732212

[CIT0008] Wei D, Qin Q, Xun F, Liu Q. 2012. EAG and behavioral response of different attractants and field trapping effect on *Asias halodendri* (Pallas). J Tian Normal Univ. 32(3):71–74.

